# Applications of Extracellular Vesicles in Triple-Negative Breast Cancer

**DOI:** 10.3390/cancers14020451

**Published:** 2022-01-17

**Authors:** Frederic St-Denis-Bissonnette, Rachil Khoury, Karan Mediratta, Sara El-Sahli, Lisheng Wang, Jessie R. Lavoie

**Affiliations:** 1 Department of Biochemistry, Microbiology and Immunology, Faculty of Medicine, University of Ottawa, 451 Smyth Road, Ottawa, ON K1H 8M5, Canada; frederic.st-denis-bissonnette@hc-sc.gc.ca (F.S.-D.-B.); rkhou085@uottawa.ca (R.K.); kmedi072@uottawa.ca (K.M.); selsa056@uottawa.ca (S.E.-S.); 2 Centre for Biologics Evaluation, Biologic and Radiopharmaceutical Drugs Directorate, Health Products and Food Branch, Health Canada, Ottawa, ON K1A 0K9, Canada; 3 Centre for Infection, Immunity and Inflammation, University of Ottawa, 451 Smyth Road, Ottawa, ON K1H 8M5, Canada; 4 Ottawa Institute of Systems Biology, University of Ottawa, 451 Smyth Road, Ottawa, ON K1H 8M5, Canada; 5 Regenerative Medicine Program, Ottawa Hospital Research Institute, Ottawa, ON K1H 8L6, Canada

**Keywords:** biomarkers, blood–brain barrier (BBB), cancer, chemotherapy, exosome, extracellular vesicles (EVs), diagnosis, nanoscale drug delivery system (NDDS), immunotherapy, nanomedicine, nanoparticles (NPs), prognosis, solid tumour, triple-negative breast cancer (TNBC)

## Abstract

**Simple Summary:**

Triple-negative breast cancer (TNBC) is the deadliest subtype of breast cancer, with limited treatment options. This review summarizes the most recent applications of extracellular vesicles (EVs) in TNBC as diagnostic/prognostic biomarkers, nanoscale drug delivery systems (NDDS) and immunotherapeutic agents, as well as the associated challenges and future directions of EV applications in cancer immunotherapy.

**Abstract:**

Triple-negative breast cancer (TNBC) is the most aggressive and refractory subtype of breast cancer, often occurring in younger patients with poor clinical prognosis. Given the current lack of specific targets for effective intervention, the development of better treatment strategies remains an unmet medical need. Over the last decade, the field of extracellular vesicles (EVs) has grown tremendously, offering immense potential for clinical diagnosis/prognosis and therapeutic applications. While TNBC-EVs have been shown to play an important role in tumorigenesis, chemoresistance and metastasis, they could be repurposed as potential biomarkers for TNBC diagnosis and prognosis. Furthermore, EVs from various cell types can be utilized as nanoscale drug delivery systems (NDDS) for TNBC treatment. Remarkably, EVs generated from specific immune cell subsets have been shown to delay solid tumour growth and reduce tumour burden, suggesting a new immunotherapy approach for TNBC. Intrinsically, EVs can cross the blood–brain barrier (BBB), which holds great potential to treat the brain metastases diagnosed in one third of TNBC patients that remains a substantial clinical challenge. In this review, we present the most recent applications of EVs in TNBC as diagnostic/prognostic biomarkers, nanoscale drug delivery systems and immunotherapeutic agents, as well as discuss the associated challenges and future directions of EVs in cancer immunotherapy.

## 1. Treatment Approaches in TNBC

Breast cancer (BC) accounts for 25–30% of all new cases of cancer in women while remaining the leading cause of death among women worldwide, accounting for 13–15% of all cases of cancer death, according to the Canadian Cancer Society (https://cancer.ca/en/cancer-information/cancer-types/breast/statistics accessed on 29 November 2021) and American Cancer Society [1]. Triple-negative breast cancer (TNBC) is the most aggressive and refractory subtype of breast cancer [2,3]. TNBC is characterized by the absence of three receptors that are commonly found in other subtypes of BC: the estrogen receptor (ER), the progesterone receptor (PR) and the human epidermal growth factor receptor 2 (HER2). As a result, TNBC is unresponsive to hormonal and/or HER2-based therapy. TNBC can be further divided into several subtypes based on gene expression profiles where the clinical outcome may differ [4,5,6,7]. For the purpose of this review, we will refer to TNBC encompassing all its subtypes. Not only is the likelihood and incidence of recurrence greater for this subset, but it also has a worse prognosis. According to the Surveillance, Epidemiology and End Results (SEER) Program (https://seer.cancer.gov/statfacts/) accessed on 29 November 2021, the five-year survival rate for TNBC is 76.9%, while for other subtypes of BC as a whole is 90.3%. This reflects the disproportional BC-related deaths associated with TNBC. Additionally, TNBC is more prevalent in women, in younger patients (<50 years old), in African American women and in individuals with a mutation in the BRCA1/2 and PALB2 genes [8,9,10]. 

Currently, surgery and radiation combined with chemotherapy and neoadjuvant therapy are the mainstays of treatment for TNBC, although novel therapeutic approaches are emerging [11]. Approved chemotherapeutics include platinum; anthracyclines, such as doxorubicin (DOX); taxanes, such as paclitaxel (PTX); antimetabolites, such as capecitabine; gemcitabine; and cytoskeletal inhibitors, such as eribulin [11]. Originally, chemotherapeutic drugs were an attractive therapeutic modality since fast-dividing cells, such as tumour cells, are sensitive to those agents. However, even if those drugs work well to some degree for cancer treatment, their application is limited. The main drawback is the dividing rate of tumour cells does not necessarily correlate with cancerous proliferation [12], thus rendering chemotherapeutic drugs suboptimal due to the enrichment of cancer stem cells, which closely associate with disease relapse and systematic side effects on the vital organs. The chemotherapeutic agents also exhibit low aqueous solubility, non-specific tumour targeting, rapid elimination in vivo and severe side effects [13]. Together, chemotherapeutics are known to have a narrow therapeutic window. Additionally, most of them are unable to cross the blood–brain barrier (BBB), which limits the treatment of brain tumours, including those arising from metastatic TNBC. About one third of TNBC patients will receive a diagnosis of brain metastasis, representing the end-stage of the disease as no cure is available to date. For these patients, the median survival has been reported as low as three to four months after diagnosis [14,15]. Furthermore, chemotherapeutic resistance and enrichment of cancer stem cells after chemotherapy are very common manifestations in TNBC patients, increasing the likelihood of metastasis and tumorigenesis [16,17]. For some individuals with BRCA1/2 mutations, they can undergo treatment using poly (ADP-ribose) polymerase inhibitor therapy using compounds such as olaparib (Lynparza) and talazoparib (Talzenna) [18]. However, the majority of TNBC patients have limited treatment options. As such, the development of novel and effective therapeutic approaches with high on-target specificity and low off-tumour toxicity is urgently needed to address the unmet medical need faced by TNBC patients.

Over the past decades, significant efforts have been made attempting to find effective alternatives to improve the outcome of immunotherapy, as well as the prognostic and diagnostic value for TNBC. Immunotherapeutic agents, capable of modulating the immune system, include unconjugated or conjugated monoclonal antibodies (including immune checkpoint inhibitors), recombinant cytokine therapy, cancer vaccine, adoptive cell transfer and oncolytic virus therapy [19]. More recently, chimeric antigen receptor (CAR)-engineered immune cells have been developed to recognize tumour cells via specific cancer cell antigens. Currently, the only FDA-approved immunotherapy for TNBC treatment is the immune checkpoint inhibitor anti-PD-1 antagonist monoclonal antibody (pembrolizumab (Keytruda)) [20,21]. In 2019, the FDA conditionally approved an anti-PD-L1 monoclonal antibody, atezolizumab (Tecentriq), for the treatment of patients with TNBC whose tumours express PD-L1 [21,22]; however, in late 2021, the manufacturer Roche withdrew its investigational therapy due to a lack of clinical benefit obtained from their phase 3 Impassion131 trial (NCT03125902) [22]. Their monoclonal antibodies are currently under investigation for TNBC, including an anti-CTLA-4 monoclonal antibody (ipilimumab (Yervoy)); however, it has shown minimal efficacy [23,24] and has yet to be approved by the FDA (e.g., active registered clinical trials NCT03546686 and NCT03818685). Additionally, one example of immunotherapy using a monoclonal antibody that does not act as an immune checkpoint inhibitor is the most recent FDA-approved sacituzumab (Trodelvy), a tumour-associated calcium signal transducer 2 (TROP2) antibody conjugated to SN-38 (topoisomerase/inhibitor chemotherapy) [25,26]. An emerging landscape of product development for cancer immunotherapy approaches is currently addressing the shortfalls and limitations, including cytokine release syndrome and immune effector cell-associated neurotoxicity syndrome, among other treatment-related adverse events. The application of immunotherapy in TNBC has been reviewed elsewhere [27,28,29,30,31,32]. Currently, thirteen active clinical trials have been registered using immunotherapy-based approaches for TNBC, where ten of those are currently in phase 1. Comparatively, fifty-five active clinical trials have been registered for TNBC using chemotherapeutic drugs, where most of them are being used either in combination or as neoadjuvant to immunotherapy/targeted therapy [33].

Cell-based therapy is also in the frontline management of cancer (reviewed in [34]). However, utilizing cell-based therapy is not without limitations and shortfalls. For example, adoptive cell transfer requires lymphodepletion in order to tilt the homeostatic balance towards the expansion of the transfused cells versus the host cells [35,36]. Comparatively, administration of therapeutic extracellular vesicles (EVs) does not require lymphodepletion, which could significantly benefit the patient. This review focuses on a cell-free immunotherapy approach for TNBC, namely, EV-based therapeutics.

## 2. The Innate Properties of EVs as Cancer Therapeutics

Over the last decade, the field of EVs has grown tremendously for many reasons, including their potential for clinical diagnosis/prognosis and their remarkable potential for therapeutic applications. EV is a collective term established by the International Society of EVs covering various subtypes of cell-derived vesicles released in the extracellular environment. Hence, the EV terminology will be used in this review. Depending on their size range and biogenesis pathway, EVs can be classified into three main categories: exosomes, microvesicles or ectosomes and apoptotic bodies [37]. Important in cell–cell communication (i.e., autocrine, paracrine, endocrine and exocrine signaling), EVs act as a biological communication system between cells that relies on receptor–ligand interactions, direct fusion with the plasma membrane and endocytosis [37,38]. The EV membrane is composed of cell-derived lipids and (glyco) proteins [37], and their content (nucleic acids, proteins and metabolites) reflects the nature and status of the EV parental cell at the time of production [38,39,40,41]. The secretion of EVs is an evolutionarily conserved process that can be accomplished by all cell types, suggesting their fundamental role in cellular physiological responses and in tissue development [42]. EVs are found in all biological fluids, such as urine, blood, cerebrospinal fluid, amniotic fluid, breast milk, seminal fluid and saliva [43,44]. In addition, they can be isolated from cell culture supernatants in a laboratory setting.

In complex organisms, including human beings, EVs exert high biocompatibility, further enhancing their bioavailability across biological barriers, including the BBB [43,45]. For example, Alvarez-Erviti et al. demonstrated the successful delivery of systemically administered EVs containing short interfering RNA specifically to the brain in mice [46]. More of these examples have been reported [47,48,49]. These intrinsic features distinguish EVs from other therapeutic agents of a similar size or nature, such as synthetic nanoparticles (NPs). For example, the presence of CD47, a receptor involved in the inhibition of phagocytosis and frequently referred to as the “don’t eat me signal”, reduces the rate of EV clearance [50]. Indeed, various surface molecules correlate with the improved lifespan of EVs in the circulation, such as CD31 (PECAM-1), CD24, β2-microglobulin (β2M), PD-L1, CD44 and dehydroxymethylepoxyquinomicin (DHMEQ), in addition to PEGylation (a typical coating process performed with NP formulations) [51]. According to the authors, the surface profile is of particular importance, and can dictate the EV half-life from minutes to hours [51]. These camouflage strategies reported to improve the EV half-life are discussed in detail by Parada et al. [51].

EV-based therapy (considered cell-free), in contrast to cell-based therapy, could avoid the severe toxicity reported with administration of cells such as cytokine release syndrome and immune effector cell-associated neurotoxicity syndrome [52]. Furthermore, while the efficacy of cell-based therapy can be altered by the immunosuppressive and acidic environment of the tumour microenvironment (TME), EVs are intrinsically unresponsive to the host tumour microenvironment as they are inert and therefore do not self-replicate. Conversely, they do not respond to signal transduction-induced modification in the same way as cells upon administration. Additionally, the EV membrane shields cargo from the environment, thus protecting the encapsulated material (e.g., nucleic acids, proteins and drugs), allowing a prolonged shelf life and increased in vivo stability of the delivered materials [53]. Their nanosized nature allows for filtration for sterilization purposes, and their continuous production in bioreactors without the need to harvest the producer cells [54] are suitable characteristics for large-scale manufacturing and as an off-the-shelf product. However promising, large-scale manufacturing of EVs is still under investigation for certain cell types since the scalability of EV production is not always feasible. On the plus side, long-term storage of EVs is achievable either by freezing procedures or lyophilization, although more research is required to determine the optimal storage conditions to maximize product stability and integrity [55,56]. The most commonly reported routes of EV administration include intravenous injections, but others have also been documented (oral, intranasal, subcutaneous, intramuscular, intrathecal, intraocular, intraperitoneal and intramyocardial [57]), rendering EVs as a versatile therapeutic tool. EVs can also be modified to express or carry molecules of interest for targeted therapeutics, opening a myriad of EV formulations and treatment plans for difficult-to-treat diseases, such as TNBC, and their associated brain metastases [58,59].

The properties of EVs generate their potential for diverse applications, including serving as potential diagnostic/prognostic biomarkers and nanoscale drug delivery systems (NDDS) in multiple cancers, including TNBC. EVs as NDDS (EV-NDDS) loaded with active compounds (e.g., chemotherapeutics, proteins and nucleotides) will increase drug penetrance, stability, solubility, lifespan and cellular uptake in the targeted sites, thereby enhancing treatment efficacy [60]. Although this may be achieved with synthetic NPs, the latter can be limited by immunogenicity [61,62], certain toxicity and the incapacity of crossing the BBB without specifically modifying their structure with functional ligands or carrier molecules [63,64]. A comparison between synthetic NPs and EVs is further discussed in Section 5.

EV-based therapeutics could potentially improve TNBC treatment efficacy and allow for the treatment of brain metastasis as well as a reduction in undesired side effects for this difficult-to-treat cancer. Many groups are currently investigating EVs as a cell-free therapeutic approach for cancer, either as a native or a bioengineered product. This novel approach for the delivery of chemotherapeutics utilizing EV-NDDS could improve the drug therapeutic index by increasing their bioavailability, improving the pharmacokinetics and overcoming some chemoresistance. This is partially because EVs are subjected to the enhanced permeability and retention (EPR) effect, resulting in their accumulation in the tumour tissue [65]. Additionally, EV-NDDS can reduce toxicity associated with conventional chemotherapeutics and increase the half-life of its carrying payload [66,67]. For example, DOX encapsulated in EVs exhibits less toxicity to allow treatment at a higher concentration and reduces the growth of MDA-MB-231 tumours in a mouse-xenograft model [68]. Furthermore, EVs engineered to target HER2-positive colon cancer cells while co-encapsulating miR-21 inhibitor with the chemotherapeutic agent 5-fluorouracil were shown to overcome chemoresistance in colon cancer [69]. This study also demonstrates the ability of engineering EVs to increase the co-delivery of multiple relevant therapeutic agents (nucleic acids and small molecules such as chemotherapeutics) to target cancer cells. Importantly, safety concerns that are accompanied by cell-based therapies, such as transplant immune compatibility, tumorigenicity potential, embolus formation and the transmission of infections (i.e., virus), could be minimized using EV-based therapies [70].

**Figure 1:**
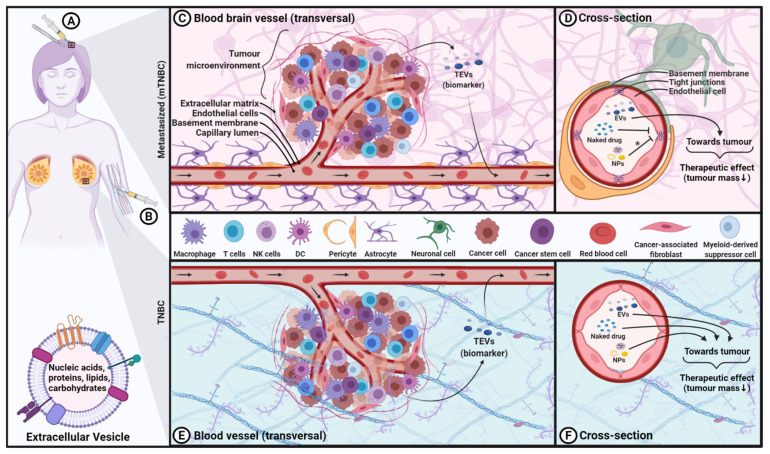
Summary of the potential applications of extracellular vesicles (EVs) in triple-negative breast cancer (TNBC). Panels (**A**,**B**) show the conventional surgery (panel **A**) for the treatment of TNBC metastasized to the brain and the proposed treatment approach via systemic administration of EVs panel (**B**). Panel (**C**) depicts a transversal view of a brain blood vessel in the tumour microenvironment of TNBC metastasized to the brain, highlighting that the TNBC-derived tumour-EVs (TNBC-TEVs) released into the blood could be used as biomarkers for diagnostic/prognostic purposes. Panel (**D**) shows a cross-section view of the same blood vessel, illustrating that only EVs are capable of crossing the blood–brain barrier (BBB) to target metastasized TNBC in the brain, but not naked drugs and conventional synthetic nanoparticles (NPs). Panel (**E**) illustrates the diagnostic/prognostic potential of TNBC-TEVs for none-metastatic TNBC in the mammary glands or TNBC metastasized to other organs outside the brain. Panel (**F**) shows that all naked drugs, synthetic NPs and EVs could cross blood vessels to TNBC tumors outside the brain. The diagnostic/prognostic biomarkers of TNBC-TEVs are listed in Table 1 and the therapeutic molecules delivered via various EV-NDDS for the treatment of TNBC are listed in Table 2. This figure was created with BioRender.com.

To summarize, EVs have inherent characteristics that make them the ideal next generation of NDDS for cancer therapies where the aforementioned properties uniquely distinguish them from other therapeutics, including cell-based therapies (see Figure 1). Granting that EVs could become new cancer therapeutic agents for TNBC treatment, more research is warranted on chemistry manufacturing and controls, as well as on efficacy and safety aspects.

## 3. Using EVs as Diagnostic/Prognostic Biomarkers for TNBC

Paradoxically, EVs can also be used as biomarkers of certain pathophysiological conditions, including cancer, since the EVs’ molecular constituents usually mirror the characteristics of their parental cells [39,44,54,71]. For example, tumour and stromal cells present in the TME were found to release EVs containing oncogenic material that was transferred to nearby cells, consequently increasing tumorigenesis, tissue invasion and metastasis, as well as stimulating angiogenesis, proliferation and immune system evasion mechanisms [72,73,74,75]. For instance, tumour-derived EVs (TEVs) from TNBC cells carrying CCL5 on their surface were shown to influence the behaviour of TME resident macrophages, rendering them pro-metastatic in nature, which ultimately led to a TME favourable for tumorigenesis [74,76]. Furthermore, TEVs are mediators of malignant transformation via Wnt signaling, modulating the cancer stem cells’ equilibrium [77]. This pathophysiological mechanism may be fed by TEVs derived from cancer stem cells [78]. With this in mind, the application of TEVs as diagnostic and prognostic biomarkers is under clinical investigation. Currently, only one clinical trial has been registered investigating this avenue (NCT04523389) for early detection of colorectal cancer; more will certainly emerge in the next decade.

One of the many pathological functions of TEVs is their ability to transfer a malignant phenotype to healthy cells and establish a fertile local and distant TME that promotes tumorigenesis and prometastatic niches, leading to metastatic TNBC [40,78,79,80,81]. TEVs may alter the transcriptome of receiving cells [82], in addition to modifying the immune response and other physiological functions, which consequently contribute to tumorigenesis and sustain tumour-related angiogenesis [74,83,84,85]. TNBC-TEVs increase the potential for pre-metastatic niche formation with distinct protein properties that proportionally increase cell motility, suggesting that TEVs are likely contributors to metastasis [86]. TEVs also facilitate chemoresistance in TNBC. For example, breast cancer TEVs (BC-TEVs) were found to export DOX into the extracellular environment and contribute to chemoresistance [87]. Besides this direct interaction with the drugs, TNBC-TEVs may have the potential to mediate the horizontal transfer of drug efflux pumps, including the ATP-binding cassette transporter between tumour cells [88]. Similarly, BC-TEVs from resistant breast MCF-7 cancer cells were shown to transfer hormones/metformin/tamoxifen chemoresistance capabilities to drug-sensitive MCF-7 cells [89]. Other examples of TNBC-TEVs transferring chemoresistance to healthy cells also exist [90]. Since TEV-mediate acquired hormonal resistance against these types of hormone-targeting therapeutics (cornerstone for treating ER+ and PR+ BC), this can be problematic and may worsen the clinical outcomes in hormone-sensitive BC [89,91]. MCF-7-EVs were also found to upregulate Wnt 5a in macrophages, which reciprocally produced EVs that enhanced tumorigenesis by enabling malignant invasion via the β-catenin-independent Wnt signaling pathway in cancerous cells [92]. BC cells under hypoxic and acidic conditions were found to upregulate EV secretion, which might contribute to tumorigenesis [73,81]. Although the BC cell line MCF-7 is non-TNBC classified because it expresses ER+PR+, it would be interesting to see if the above observations apply to TNBC.

While TEVs have been reported and reviewed extensively for their role in many pathologies, they are also considered as diagnosis/prognosis biomarkers for TNBC after being repurposed [40,44,71,93,94]. There is of great interest in early detection of solid cancer via lesser invasive medical interventions. For example, EVs can be retrieved from liquid biopsies, which include biological fluids such as serum, urine, ascites fluids and pleural effusion [37,40,95], and analyzed with the appropriate method to establish a diagnosis. This is possible because of their systematic presence in most physiological fluids, prominent stability in circulation and the fact that they mirror their parental cell phenotypic profile and molecular characteristics [38,96]. In other words, TNBC-TEVs will reflect the phenotypic traits and molecular pathology of the parental TNBC cells, thereby bearing a TNBC-specific cargo that can be used as diagnostic/prognostic biomarkers. In turn, TEVs can inform on the cancer molecular pathological state to develop future drug treatment [73]. One study even reveals how these non-invasive liquid biopsies can discriminate between the different subtypes of BC based on the distinctiveness of the TEV-proteome and metabolome [97,98]. Indeed, many studies have identified miRNAs in TEVs that can be used as biomarkers for the diagnosis/prognosis of TNBC, among other types of BC [99]. MicroRNA (miRNA/miR) are small (roughly 20 nucleotides long) non-coding RNA that regulate gene expression at the post-transcriptional level. Their functional role has been linked to a diverse range of pathophysiological effects, such as the acquisition of chemoresistance, reduction in chemosensitivity, metastatic potential and vascular permeability and promotion of tumorigenesis [100]. Many miRNAs are involved in BC pathophysiology where TEVs transfer miRNAs from one cell to another, affecting epithelial–mesenchymal transition (miR-105, miR-122 and miR-939), tumorigenesis, invasion and metastasis (let-7a miRNA, miR-21, miR-92b, miR-130a, miR-149, miR-181c, miR-200, miR-328, miR-423-5p, miR-602 and miR-1246) and the transfer of chemoresistance abilities (miR-21, miR-221/222, miR-423-5p, miR-770 and miR-1246) [100,101,102]. Similar functional miRNAs (miR-17, miR-30a, miR-100 and miR-222), contributing to the horizontal transfer of cargo, allowing for chemoresistance, were found in MCF-7-EVs [103]. The miRNA pattern obtained from the TEVs derived from HER2+ BC and TNBC was shown to be different, implying an important feature of precise subtype identification among the different BC subtypes [104]. TNBC-TEVs encapsulating known RNA species as cargo with diagnosis/prognostic purposes are summarized in Table 1.

**Table 1:** TNBC-TEVs encapsulating known RNA species and protein cargo with potential diagnosis/prognostic value.

Cargo	Parental Cell Origin	Reported Effect and Outcomes in TNBC	Refs.
RNA Species			
let-7a miRNA, miR-328, miR-130a, miR-149, miR-602, and miR-92b	MDA-MB-231	Promotes tumorigenesis, invasion and metastasis	[80]
miR-21 and miR-1246	TNBC and BC patient serum	Promotes invasion, metastasis and chemoresistance	[105,106]
miR-27b, miR-335, miR-376c, miR-382, miR-433, and miR-628	TNBC patient serum	Various effect; high-throughput screening for miRNAs in TNBC-TEVs	[104]
miR-7e, miR-10b, miR-32, miR-106b and miR-138	MDA-MB-231	Promotes invasion and metastasis	[107]
miR-101 and miR-373	TNBC patient serum	Downregulates ER expression and inhibits camptothecin-induced apoptosis	[108]
miR-105	MDA-MB-231	Promotes invasion and metastasis by specifically targeting tight junction protein Promotes angiogenesis	[109]
miR-122	MDA-MB-231	Promotes metastasis and the establishment of a pre-metastatic niche	[110]
miR-134	Hs578T	Reduces cancer aggressiveness and increases drug sensitivity	[111]
miR-137 and miR-496	MDA-MB-231	Promotes proliferation and invasion	[112]
miR-181c	MDA-MB-231-luc-D3H2LN	Promotes invasion and metastasis by disrupting the integrity of the BBB	[113]
miR-200	MDA-MB-231	Promotes metastasis and the establishment of a pre-metastatic niche	[114]
miR-223	Macrophages	Promotes invasion via a positive feedback loop using EV communication platform	[115]
miR-423-5p	MDA-MB-231	Promotes chemoresistance	[116]
miR-770	MDA-MB-231 MDA-MB-468	Suppresses the DOX-resistance mechanism	[117]
miR-939	MDA-MB-231	Regulates VE-cadherin in endothelial cells, which enhances cancer cell’s trans-endothelial migration	[118]
circPSMA1	MDA-MB-231	Facilitates tumorigenesis, metastasis and migration via miR-637/Akt1/β-catenin (cyclin D1) axis	[119]
lncRNA XIST	TNBC patient serum	Increases tumour recurrence	[120]
Proteins			
UCHL1	Various TNBC cell lines, various PDX, TNBC patient-serum	Stimulates migration, extravasation and promotes tumor progression	[121]
CD151	MDA-MB-231 and TNBC patient-serum	Stimulates migration and invasion	[122]
EGFR	MDA-MB-231	Stimulates invasion	[123]
Survivin	MDA-MB-231	Promotes tumour survival	[124]

MDA-MB-231 and Hs578T are TNBC (ER-PR-HER2-) cell lines; MCF-7 is an ER+PR+HER2- cell line; UCHL1: ubiquitin carboxyl-terminal hydrolase isozyme L1; PDX: patient-derived xenografts; EGFR: epidermal growth factor receptor.

Although informative, the majority of the preclinical studies described in Table 1 relies on TNBC/BC cell lines, which may not reflect the true nature of patient BC-TEVs, therefore warranting further investigations using patient-derived materials [125,126]. Additionally, clinical trial results would inform the validity of known RNA species from TNBC-TEVs’ cargo as diagnostic/prognostic biomarkers, but such investigation still awaits to be conducted. This may address the reproducibility concern across studies using the same cell line (e.g., MDA-MB-231) where different miRNA profiles were reported. While most studies reported miRNAs as biomarkers in TEVs, other biological materials delivered via TEVs can serve as biomarkers for TNBC. For example, UCHL1 (ubiquitin carboxyl-terminal hydrolase isozyme L1) harboured in TNBC-TEVs has been proposed as a biomarker for TNBC [121]. It is also applicable to use surface membrane proteins (e.g., CD151) present on TNBC-TEVs as biomarkers for diagnosing TNBC [122]. Additional examples of potential protein biomarkers for TNBC-TEVs include the epidermal growth factor receptor (EGFR) and survivin [123,124,127]. Thus, a large repertoire of biological molecules can be harnessed from TEVs to gain diagnostic/prognostic information.

From a cellular perspective, tumour-infiltrating lymphocytes (TILs), a key player with increasing popularity due to their ability to control tumour growth in the TME, have emerged as prognostic markers in TNBC [79,128,129]. Indeed, there is evidence that an elevated rate of TIL results in improved TNBC and HER2+ BC outcomes [130]. Yeong et al. reported that infiltrating the CD8^+^/PD-1^+^ immune subset was associated with improved disease-free survival in TNBC, suggesting a prognostic value for TNBC [130]. The adoption of PD-L1 as a prognostic biomarker of BC has been reported where PD-L1 expression in tumour cells correlates positively with the overall survival and disease-free survival in TNBC [130,131]. Unfortunately, a gap in the literature about TIL-EVs derived from TNBC patients currently exists. Filling this gap could bring novel diagnostic/prognostic biomarkers for TNBC and maybe even open a potential therapeutic avenue [128,132,133]. From an adoptive cell transfer perspective, applying autologous TILs for cancer treatment brings interest, with currently only one clinical trial dedicated towards TNBC (NCT04111510; phase II).

## 4. Novel Treatment Avenues Using EVs for TNBC

Earlier in the review, we have introduced the latest immunotherapy treatment strategies for TNBC. Among the panel, EV-NDDS seems to be a promising approach, especially when combined with the aforementioned immunotherapeutic agents [134]. EVs are naturally occurring carriers of endogenous materials (such as nucleic acids, proteins and lipids) and can be further manipulated in the laboratory as EV-NDDS to encapsulate exogenous materials (such as small molecules) to be delivered to the desired target site. One recent study showed active encapsulation of soluble molecules using a light-inducible loading system called “EXPLOR” that is complexed with a fusion protein system, resulting in a 40-fold enrichment of the desired material within the EVs [135]. Many similar EV-NDDS strategies exist, allowing for the delivery of a broad range of therapeutic agents to their desired target sites, as long as the cargo remains functional once delivered. In any case, enriching an EV population to contain more or less of a cargo type directly depends on the EV source, method of isolation and any exogenous loading method or engineering approach, if required [59,70,136]. Additionally, it is increasingly evident that EVs play numerous roles that are typically dependent on the parental cell from which they are derived. Effectively, administered natural killer (NK) cell-derived EVs (NK-EVs) were shown to arrest the tumour progression of melanoma and neuroblastoma by enhancing the NK cells’ anti-tumour immunity, thus demonstrating the potential application of native NK-EVs as a novel cancer immunotherapy modality [137,138]. Thus, by exploiting the exchange of information between EVs and the TME, it is possible to modulate the anti-tumour immune response to prevent metastasis and hinder tumorigenesis [41]. In fact, EVs derived from a panel of immune cells, such as NK cells, CD8+ cytotoxic T cells (CTLs), dendritic cells (DCs) or CAR-engineered immune cells, have been shown to interact with cancer cells, where they can induce various immune responses [139].

In this section, we cover reports describing the EVs derived from various cell types that have therapeutic potential for the treatment of TNBC based on their cargo, including small molecules, nucleic acids and membrane-embedded molecules. We also cover the therapeutic relevance of the EVs derived from cytotoxic immune effector cells and the EVs used as a cancer vaccine for TNBC. Cargo encapsulated in EV-NDDS for TNBC treatment is summarized in Table 2.

**Table 2:** Cargo encapsulated in EV-NDDS for TNBC and BC treatment.

Cargo Type	Cargo	Parental Cell	Receiving Cell	Reported Effect and Outcomes	Refs.
Small molecules	DOX	Immature DCs	MDA-MB-231 MCF-7	Inhibiting tumour growth without overt toxicity	[140]
	DOX	MDA-MB-231 and STOSE ovarian cancer	MDA-MB-231 and STOSE ovarian cancer	EV-DOX is less toxic and allows treating mice at a higher concentration, reducing the volume of the tumour.	[68]
	PTX and DOX	Macrophages	MDA-MB231	Enhancing anti-proliferation effect	[141]
	Curcumin	B16 (melanoma), TS/A (adenocarcinoma), and 4T.1	NK cells	Restoring the strongest effect to the cytotoxic function of NK cells	[142]
	β-Elemene	MCF-7	MCF-7/Docetaxel MCF-7/Adriamycin	Significantly reversing the BC chemoresistance	[143]
	Erastin	HFL-1 (normal human lung fibroblast)	MDA-MB231	Robust accumulation of erastin and increasing killing effect	[144]
Nucleic acids	TPD52-siRNA	HEK293T	SKBR3 MDA-MB231	siRNA downregulation of gene expression by 70% of cancer cells although no conclusion was drawn on the effect of this silencing	[145]
	VEGF-siRNA and let-7a miRNA	Primary DCs	MDA-MB-231	Selectively targeting nucleolin-positive tumour tissues and inhibiting tumour growth	[146]
	let-7a miRNA	HEK293 cells	HCC70	Significantly inhibiting tumour growth	[66]
	miR-127, miR-197, miR-222, and miR-223	Bone marrow stroma	MDA-MB-231	Enhancing anti-proliferation effect	[147]
	miR-142-3p inhibitor	MSCs	4T1 (mouse)	Efficiently delivering anti-miR-142-3p and restraining cancer proliferation	[148]
	miR-9 and miR-155	MDA-MB-231	MCF-7	Remarkably downregulating PTEN and DUSP14 in tumour cells	[149]
	miR-381	MSCs	MDA-MB-231	Reducing cancer metastatic behaviours	[150]
	miR-496	MCF10A	MDA-MB-231	Exerting a tumour suppressive role by targeting Del-1	[112]
	miR-424-5p	Adipose tissue-derived-MSCs	MDA-MB-231	Promoting apoptosis of TNBC by suppressing PD-L1 signaling	[151]
Membrane-embedded molecules	Human IL-3Rα/CD123 Mab	Human TEC (thymic epithelial cells)	MDA-MB-231	Reducing cell viability and cell migration	[152]
	HER2	BT-474	MDA-MB-231	Receptor HER2 conferred on the surface of TNBC cells allowing for anti-HER2 antibody delivery therapy	[153]

Mab: monoclonal antibody; MDA-MB-231, Hs578T, HCC70 and 4TI (mouse) are TNBC (ER-PR-HER2-) cell lines; MCF-7 and SKBR3 are ER+PR+HER2- cell lines; MSCs: mesenchymal stromal/stem cells; PTX: paclitaxel; DOX: doxorubicin.

### 4.1. TNBC Treatment with EV-NDDS Containing Therapeutically Relevant Small Molecules

Many small therapeutic molecules (e.g., doxorubicin, paclitaxel, curcumin, erastin, β-elemene) used in cancer treatment have shown efficacy, although their therapeutic effect can be limited by low bioavailability, plasma instability, low accumulation within the target site or/and high off-target toxicity [13,66,67,154]. Therefore, drug encapsulation strategies are of high interest as a solution to overcome these barriers. For their numerous appealing intrinsic properties, EV-NDDS are surging across research as the next generation of NDDS. Encapsulation of exogeneous materials can be performed post-EV isolation via sonication, extrusion, electroporation, freeze-thawing or drug conjugation techniques [58,155]. Alternatively, passive drug encapsulation involves drug diffusion into EVs or drug uptake by donor cells and subsequent secretion of drug-encapsulated EVs; although, one would have to ensure that the drugs utilized in passive encapsulation will not impact the viability of the EV-producing cells.

In BC treatment, Hadla et al. demonstrated the importance of the EVs isolated from the MDA-MB-231 TNBC and STOSE ovarian cancer cell lines for DOX delivery with reduced cytotoxicity, particularly heart toxicity [68]. Additionally, Gong et al. have shown the synergetic effect of the EVs encapsulated with DOX and miR-159 against MDA-MB-231 TNBC cell lines [156]. Furthermore, the administration of immature DC-EVs encapsulating DOX suppressed the growth of MDA-MB-231 tumours in nude mice [140]. This tumour growth suppression effect was also confirmed in a zebrafish cancer model, where the chemotherapeutic agents PTX and DOX encapsulated in EVs were delivered across the BBB for the treatment of brain metastatic TNBC [157]. Additionally, macrophage-EVs encapsulating PTX and/or DOX have been shown to be delivered and act successfully against TNBC [141]. These results are promising since TNBC exhibits a high incidence of brain metastasis where the application of EV-NDDS provides a glimmer of hope for improvement of clinical outcomes in patients diagnosed with brain tumours [158].

### 4.2. TNBC Treatment with EVs Containing Therapeutically Relevant Nucleic Acids

EVs naturally carry various types of RNA molecules, such as miRNAs that regulate post-transcriptional modifications, that can have multiple effects on receiving cells, including tumour suppression [159,160,161]. Most RNA species, including small interfering RNA (siRNA), that have shown clinical benefit by disrupting genes of interest, tend to have low stability in the systemic circulation and rapid degradation if injected without a carrier, due to their short half-life [162]. Onpattro (patisiran), an approved lipid nanoparticle containing an RNA interference (RNAi)-based drug for the treatment of polyneuropathy of hereditary transthyretin-mediated amyloidosis in adults, is a good example of a siRNA encapsulation strategy for RNAi therapy [163]. Similarly, EV-NDDS is highly considered for gene therapy applications, due to the protective environment provided by the EV lipid bilayer membrane against enzymatic degradation and the potential to direct EVs to specific tissue. For instance, Wahlgren et al. reported that EVs derived from human plasma could deliver siRNA into human mononuclear blood cells [164]; this overcame the aforementioned limitations. The delivery of siRNA encapsulated in EVs was also shown to regulate post-transcriptional gene silencing and to initiate cell death in various recipient cancer lines [73]. VEGF-siRNA and let-7 miRNA encapsulated in primary DC-EVs, modified to target nucleolin via the DNA aptamer AS1411, was shown to inhibit MDA-MB-231 tumour growth [146]. Similarly, EVs derived from the bone marrow stroma encapsulated several miRNAs (miR-127, miR-197, miR-222 and miR-223) that arrested proliferation of the TNBC cell line MDA-MB-231 [147]. Furthermore, let-7a miRNA-encapsulated in engineered EVs derived from HEK293 cells can effectively be delivered to HCC70 TNBC cells implanted in Rag2-/- mice via the targeting of the epidermal growth factor receptor [66]. O’Brien et al. have demonstrated the reduction of the anti-apoptotic protein expression of Bcl-2 (via Hsp90 inhibition) and cisplatin chemoresistance following in vitro treatment of miR-134 encapsulated in EVs against the TNBC cell line Hs578T [111]. Mesenchymal stromal/stem cells (MSC)-EVs have high therapeutic relevance for various applications. For example, adipose tissue-derived MSC-EVs were shown to deliver miR-424-5p to the MDA-MB-231 TNBC cell line, increasing apoptosis of the cancer cells [151]. In line with these findings, Shojaei et al. showed that MSC-EVs encapsulated with miR-381 were able to reduce the metastatic behaviours of MDA-MB-231 TNBC cells after co-culture [150]. The authors concluded that the decrease in viability, migration and invasion of TNBC cells treated with miR-381 encapsulated in MSC-EVs occurred via the downregulation of the Wnt signaling pathway and epithelial–mesenchymal transition transcription factors [150]. It was shown that these MSC-EVs significantly downregulated Twist and Snail expression—two transcription factors involved in epithelial–mesenchymal transition in TNBC cells [150]—evidence supporting the reduction in metastatic behaviour post-treatment [165].

### 4.3. TNBC Treatment with EVs Containing Therapeutically Relevant Membrane-Embedded Molecules

Membrane-embedded molecules on EVs can be used directly or indirectly against a target cell. For example, Lopatina et al. used EVs embedded with an antibody against IL-3 receptors whereupon delivery to the MDA-MB-231 TNBC cell line expressing IL-3 receptor directly reduced the cell viability and cell migration [152]. Comparatively, EVs’ ability to transfer their membrane-embedded molecules to the recipient cell membrane upon fusion can confer the recipient cells with transiently selective receptors or antibodies for targeted therapy [166,167,168]. For example, Quinn et al. demonstrated a method through which the receptor HER2 can be conferred on the surface of MDA-MB-231 TNBC cells (HER2-) using HER2-expressing EVs derived from BT-474 breast cells overexpressing HER2 and ER [153]. Doing so allowed the targeting of TNBC cells through anti-HER2 antibody delivery [153]. These findings suggest that EVs can confer relevant receptors on tumour cells, which may promote immune cell-mediated tumour killing via targeted drug delivery. Such an EV targeting approach utilized to prime the tumour cells for targeted therapy could improve clinical outcomes. That being said, the clinical feasibility, practicality and safety aspect of transferring targetable receptors on cancerous cells remain challenging as numerous variables may hinder this approach.

### 4.4. TNBC Treatment with EVs Derived from Cytotoxic Effector Cells (NK and CD8+ T Cells)

Cytotoxic immune effector cells, such as NK and CD8+ cytotoxic T lymphocytes (CTLs), have unique immunological properties that can be exploited for cancer immunotherapy. However, effector cell-based therapy can be limited by tumour penetrance and therefore have limited efficacy under certain circumstances. With the premise that EVs derived from such immune cells would retain the cytotoxicity activity against tumour cells akin to their parental origin, while increasing their tumour penetrance, researchers are investigating the efficacy of NK-EVs and CTL-EVs for cancer treatment. The evidence suggests that immune cell-derived EVs can suppress tumour growth and metastasis of FasL+ tumour cells [169,170]. Indicatively, administered immune cell-derived EVs can interact with the cancer cells directly or can interact directly with host immune cells to regulate their anti-tumour activity to mitigate the cancer [73].

Cancer-specific T cells can recognize tumour-associated antigen (TAA) expressed on cancerous cells. Other alternative recognition mechanisms may involve genetic modification of T cells to treat cancer by providing antigen specificity [171]. Importantly, CAR-T cells have been utilized in clinical trials for TNBC immunotherapy, combining the property of antibody–antigen specificity with the effector function of T cells [30]. A recent review article summarizes the novel players used in antigen-directed CAR-T cell therapy for TNBC [30]. Thus, further research in identifying novel antigenic targets in TNBC is required to increase the repertoire of targetable receptors on TNBC to improve treatment efficacy. Harnessing EV cytotoxic potential from CTLs or engineering CAR-T or CAR-NK cells has great potential for TNBC. In line with this concept, the EVs derived from T cells harbouring PD-1 showed a multifunctional role against TNBC [131]. In this study, Qiu et al. showed that PD-1-expressing EVs derived from T cells educated the host T cells and enhanced their cytotoxic activity against the cancer cells. This interaction was mediated via the PD-1/PD-L1 axis, where the EVs prevented the attenuation of T cell cytotoxic activity. Additionally, the EVs were internalized by the cancer cells, resulting in a significant decrease in expression of surface PD-L1. This example demonstrates the direct and indirect mode of action of T cell-derived EVs as an immunotherapy approach against TNBC at the cellular and molecular levels.

Unlike CTLs, NK cells lack antigen-specific receptors and do not require major histocompatibility complex I (MHC-I) recognition to exert their cytolytic activity. NK cells are cytotoxic lymphoid cells involved in tumour immunosurveillance as part of the innate immunity, where they combat cancer metastasis, growth and tumour progression [41]. Upon detecting the tumour cells, NK cells become activated via a repertoire of inhibitory and activating receptors that bind various ligands on the tumour cells without prior antigen sensitization. In line with their innate cytolytic nature, NK-EVs have shown representative phenotypical expression profiles (i.e., CD56) and content (e.g., FasL, perforin, granzyme, etc.), reflective of NK cell biology [172]. Functionally, NK-EVs also display anti-tumour cytokilling activity towards many cancer cell lines: Jurkat (48.9%), K562 (20%) and DAUDI (37.1%) [172]. Although not shown in TNBC, injection of NK-EVs resulted in reduced tumour growth of melanoma cells, thus demonstrating their cytotoxic potential against solid tumours [137]. NK-EVs can eliminate cancer cells by various mechanisms: via caspase-independent and caspase-dependent cell death pathways and via killer proteins (granzyme A and B, perforin, FasL and granulysin) [173]. Therefore, further research targeting TNBC using NK-EVs may hold therapeutic potential.

Conclusively, the body of knowledge related to the application of EVs derived from cytotoxic immune cells is still limited because it is such an early and novel therapeutic approach. As such, many groups, including ours, are currently investigating the application of EVs derived from cytotoxic immune cells against TNBC, for which more research is warranted.

### 4.5. TNBC Treatment with EV-NDDS-Based Cancer Vaccines

Cancer vaccination is different from “normal” vaccination in its application and purpose. Normally, the goal of vaccination is to immunize an individual against a certain type of invader (bacterium, virus, etc.) so that upon encountering it in the future, the likelihood of an infection would be lowered (e.g., a vaccine against the seasonal flu). On the contrary, the goal of a cancer vaccine is to redirect the immune system towards cancer rather than prevent it. In other words, most cancer vaccines are therapeutic rather than prophylactic treatments. An exception is the prophylactic administration of a vaccine against oncoviruses (viruses causing cancer) to prevent/reduce the occurrence of cancer (e.g., the human papillomavirus (HPV) vaccine, Gardasil, FDA approved in 2006; not an EV-based platform). Thus, the idea behind immunizing an individual against an oncovirus is nearly the same as preventing any other infectious disease requiring a vaccine. Immunizing an individual with a prophylactic cancer vaccine aims to reduce cancer prevalence by triggering a specific immune response that will target cancer before it can develop. Therefore, it would appear more applicable to conceive prophylactic EV-based cancer vaccines against virally induced cancer, such as HPV, and to produce EV-based therapeutic cancer vaccines against non-virally induced cancer, such as TNBC.

EV therapeutic approaches are now surfacing as a nanoscale cancer vaccine strategy, since they mediate intercellular communication between immune and cancer cells [139,174]. This concept has been demonstrated by administering NK-EVs, which primed the host NK cells, generating a more potent cytotoxicity effect against tumour cells [138]. Three cell sources for EV-based cancer vaccines are covered in this review: DCs, NKs and tumour cells. However, EV-based cancer vaccines derived from MSCs [175] and macrophages [176] have also been reported. Interestingly, TEVs might serve as a potential EV-based vaccine platform, but present important safety considerations as they are known to contain oncogenic materials. Thus far, EV-NDDS carrying TAAs (DNA, mRNA or the protein version of the TAA) derived from normal cells is a potential substitute for TEVs [73]. Specifically, DCs that were pre-exposed to cancer peptides permitted a DC-EV cytotoxic immune response against mammary adenocarcinoma tumour cells in vivo [177]. Mechanistically, DC-EVs can prime and activate cytotoxic CTLs and NK cells, rendering them more efficient at shrinking the various tumours [139]. Various mode-of-actions have been proposed, such as direct activation, antigen transfer to nearby antigen-presenting cells (APCs) (either by membrane fusion or endosomal processing) and fusing with tumour cells, rendering them more immunogenic. Utilizing a primed DC-EV-based cancer vaccine has potential as an immunotherapeutic agent, where these EVs would educate the immune system by presenting cancer-related peptides or TAAs, thus improving the anti-cancer activity of the patient’s immune system. DC-EVs have already proven to be feasible and safe in a phase I clinical trial using autologous exosomes pulsed with melanoma-associated antigen 3 peptides for the immunization of stage III/IV melanoma patients [178]. NK-EVs have shown potential in activating certain cell types of peripheral blood mononuclear cells (PBMCs), improving their anti-cancer activity [172]. For example, NK cells previously exposed to neuroblastoma secrete EVs displaying NK cell receptors (such as CD56, NKG2D and KIR2DL2) capable of educating host NK cells, which can suppress the neuroblastoma cells in vivo in nude mice [138,179]. Federici et al. suggest that NK-EVs induced the upregulation of CD25 (a T cell activation marker) in T cells, which caused a decrease in PD-1 expression in the cells, thus rendering T cells less susceptible to repression by PD-L1 overexpressed in cancerous cells [180].

TEVs contain a variety of cytosolic and membranous TAAs, along with other endogenous danger signals such as heat shock proteins (HSC70 or HSP90) and MHC-I molecules, rendering them potential candidates for an EV-based cancer vaccine [40,139,181]. For example, Wolfers et al. activated DCs via TEVs, allowing a specific CTL activation and CTL-dependent cross-protection with different tumours, which shared TAA [181]. Furthermore, their TEV-activated DCs rejected autologous tumours in the mammary carcinoma mouse model [181]. Thus, a TEV-based cancer vaccine can contain shared tumour-rejection antigens, efficiently taken up and cross-presented on DCs by MHC-I molecules for cancer treatment [181]. However, the application of TEVs as cancer vaccines could pose some important safety risks since their inherent function is primarily to promote tumorigenesis [73,74]. Maybe a derivative of TEVs could be a safer alternative as cancer vaccines, such as EV-NDDS derived from a healthy producer cell line encapsulating either TAA’s DNA, mRNA or the protein.

Long-lasting antigen-specific immunity remains the challenge of vaccination in cancer patients to protect healthy host cells from tumour formation and metastasis [177]. Zitvogel et al. demonstrated that a single injection of DC-EVs loaded with tumour peptides induced an immune response, resulting in tumour eradication via T cell-mediated anti-tumour effects [177]. However, further research is required for the EV-based cancer vaccine strategy to enhance a long-lasting immune response against tumour cells. A strong correlation between TNBC-TEVs and TNBC-specific biomarkers would accelerate the development of an effective cancer vaccine against TNBC. Currently, six active clinical trials investigating cancer vaccines have been registered for TNBC with either chemotherapy drugs or immunotherapy agents, as described in Table 3.

## 5. EVs versus Synthetic NPs

The nanomedicine field is evolving rapidly, with ongoing research and development focused on improving the delivery of targeted therapeutics for tailored treatments and personalized medicine. Among those nanomedicine products, synthetic NPs have been revolutionary by paving the way forward as they enhance the delivery and bioavailability of chemotherapeutic drugs and improve their immunotherapy efficacy [182,183,184]. The term nanoparticle is defined by the International Union of Pure and Applied Chemistry as a particle of any shape falling in the nanometre scale [185]. Interestingly, small EVs fall into the nanosized range (<100 nm or <200 nm for small EVs, according to the minimal information for studies of extracellular vesicles (MISEV) 2018 guidelines); however, they contrast with synthetic NPs as they are biologically derived from cells and not synthetically made [37]. Indeed, therapeutically relevant NPs are nearly always artificially produced and synthesized using wet chemistry methods (referred to as synthetic NPs in this review), with the exception of viruses and virus-like particles (VLP) (such as plant-based particles), which would be considered non-synthetic NPs. In contrast, EVs are isolated from biological fluids (urine, saliva, serum, etc.) released by cells or obtained from cell culture medium in the laboratory setting and therefore display innate biological diversity [186]. Consequently, the term “synthetic” will be placed in front of NPs (i.e., using wet chemistry) to distinguish the NPs accurately at play. Mainly three classes of synthetic NPs are known: polymeric NPs (e.g., polymersome, dendrimer), inorganic NPs (e.g., silica, iron oxide) and lipid-based NPs (e.g., liposomes, micelles) [187]. Conversely, synthetic NPs range in size, in material composition (metal, lipids, polymers, etc.), in their 3D configuration (sphere, rod, etc.) and in the type of coating on their surface (functional group presentation, antibody presentation, etc.) [183,188]. Their nanoscale size renders them able to interact with the cell surface and intracellular targets without modifying their properties and behaviours [184]. Polymeric synthetic NPs are biodegradable and commonly coated with a hydrophilic polymer, polyethylene glycol (PEG), to improve the circulation lifetime and biodistribution [184,189,190,191]. Therefore, synthetic NPs can encapsulate hydrophobic drugs to increase the compound’s bioavailability without increasing the dosage. However, an immune response is mountable against PEG, limiting the repetitive administration of PEG-coated synthetic NPs [61,62,187,192]. For that reason, other molecules are under evaluation as alternatives over PEG to extend the circulation lifetime while keeping immunogenicity at bay [191]. The pharmacokinetic properties of the synthetic NP-NDDS can be optimized by formulating different combinations of particle size, dosage, surface properties and the timely release of therapeutic agents to specific sites. As such, successful synthetic NP-NDDS often have a high drug-loading capacity to reduce the number of matrix materials required for administration [183], combined with efficient drug loading and drug release characteristics and should not be immunogenic unless used as a vaccine. The reported administration routes of synthetic NPs include various parenteral routes, such as transdermal (also including intraocular), inhalation and injection, in addition to enteral routes, mainly oral [190,193,194,195].

Synthetic NP-NDDS can be manufactured to address some of the clinical challenges faced with BC treatment [196]. For example, Abraxane (a formulation of albumin-bound paclitaxel) and Doxil/Myocet/Lipo-Dox (a formulation of liposomal doxorubicin made by different manufacturers) have been approved by the FDA for the treatment of BC [197]. Indeed, synthetic NPs accumulate and get trapped in the tumour because of the abnormal vasculature and poor lymphatic drainage of the tumour-induced angiogenesis. The EPR effect, previously described, permits synthetic NPs to be utilized as a NDDS where they would target tumour cells while minimally impacting other organs simply by remaining near the tumour [198,199]. Their surfaces can be modified to incorporate functional domains to regulate their interactions with target cells [190]. They can also be programmed to release agents and compounds of interest (i.e., chemotherapy drugs) within the local environment of their target cells [188,200]. Most recently, synthetic NPs encapsulating tumour antigens, immune adjuvants and cytokines have shown efficacy in clinical trials [188], demonstrating their application as NDDS. However, most synthetic NPs cannot transport drugs across the BBB through the systemic circulation without incorporating the ligands (e.g., apolipoprotein E) necessary for receptor-mediated uptake [201]. Thus, engineering synthetic NPs by adding specific ligands can improve their clinical application for brain diseases and disorders [201]. Among all the known, therapeutically relevant synthetic NPs, liposomes are the most similar to EVs due to their morphological and materialistic structure (lipid-based) resemblances. With modifications to their surface with free ligands, liposomes can be synthesized to cross the BBB and circumvent the aforementioned limitation [202]. The comparison between naked drug delivery, synthetic NP delivery and EV delivery systems has been reviewed elsewhere [70,203,204,205,206,207]. The similarities and differences between EV-NDDS, NP-NDDS and naked drug delivery systems are highlighted in Table 4. 

It appears that nanomedicinal products offer a great improvement over naked drug delivery, which can either be achieved by the well-established synthetic NP-NDDS or the biologically derived newcomer EV-NDDS. The two types of nanoscale delivery systems present similar characteristics, as described in Table 4, with some differences. To summarize the comparative description from Table 4, EV-NDDS’s molecular composition and physicochemical properties rely on the parental cell that secretes them, whereas synthetic NP-NDDS’s properties rely on the synthesis strategy, thereby yielding uniquely engineered nanomedicinal products. The favourable properties of EVs as an NDDS were described earlier in Section 2. The synthetic NP-NDDS’s complexity and various capabilities appear to depend on the degree of control of the synthesis process [187]. For example, earlier formulations of synthetic NPs were generally simpler than current formulations. For instance, current synthetic NP formulations require additional modifications and engineering, resulting in increased stability and biocompatibility of the NP material upon administration [217]. Ultimately, this reduces the rapid clearance of the active drug and improve the extravasation of the NP-NDDS through biological barriers, thereby improving the pharmacokinetics of the treatment [213]. For example, overcoming the mononuclear phagocyte system (MPS) could improve the pharmacokinetics properties of synthetic NP-NDDS by reducing the accumulation of synthetic NPs in the lung, liver and spleen [204,228]. This concept also applies to EV-NDDS, where overcoming the MPS may reduce the dosing required. While no clinical trials conducted for TNBC using EV-NDDS have been registered yet, sixteen clinical trials are registered using synthetic NPs.

In conclusion, NP-NDDS have paved the way for nanomedicine since 1995, when the first synthetic NP-NDDS (Doxil; liposomal doxorubicin) has been approved by the FDA, originally indicated for AIDS-related Kaposi’s sarcoma; this following review covers the history of Doxil’s approval [229]. Since then, the field has been growing exponentially, where numerous NP-based drugs have been approved on the market for several applications. More recently, we have seen a rise in the EV-NDDS literature for similar applications as NP-NDDS, thus increasing the pool of future nanomedicinal products available for the delivery of various drugs to target sites of interest (e.g., solid tumour microenvironment).

## 6. Future Directions of EV Application as Therapeutics and NDDS

Evidence presenting the role of EVs in TNBC provides a further opportunity for research, particularly for the development of EVs as diagnostic/prognostic biomarkers, but more importantly, as therapeutic agents and NDDS. EVs as cancer vaccines and drug carriers have shown promising results in both preclinical and clinical trial studies, suggesting a further expansion of this research to be used as a therapeutic agent. The aforementioned properties describing the uniqueness of EV-NDDS as a cell-free therapeutic tool render it interesting for clinical applications. An important safety consideration regarding EV–NDDS when derived from immortalized or cancer cells is the possibility to carry over some oncogenic materials [70]. However, this risk can be mitigated by screening for oncogenic/viral materials prior to administration. Furthermore, it remains unclear whether frequent administrations of allogenic EVs will trigger adverse immune reactions, warranting further studies. Despite all those sought features, EV utilization remains limited because of the current challenges faced with protocol standardization, ranging from EV isolation, purification and characterization of bulk EV preparations or EV subtypes, which may differ in subcellular site of origin, size and protein identity markers, for example, as well as the specific cargo loading methodology and efficiency [37]. Towards industrial and commercial scale-up and scale-out of EVs for high-yield production, the introduction of quality controls, compliance with good manufacturing practices (GMP) and regulatory guidelines will be required for ease of clinical translation of EV-based therapies, which will remain an active research topic over the next decade. The limitations associated with the clinical application of EVs have been reviewed elsewhere [51,222,230].

On the other hand, one redundant trend in the cancer literature is the need for cancer-specific biomarkers, which are hard to find. This significant challenge remains central in TNBC. Overcoming this issue may allow for personalized treatment plans for the patients and would also allow for more effective treatments with ideally fewer side effects related to drug toxicity [78]. With our current understanding of general cancer treatment, a good and successful treatment plan absolutely and undeniably requires a combinatorial approach. This mainly arises from the complex heterogeneity of cancer, even within one patient. The goal is to eliminate cancer in a more complementary and synergistic approach to reduce the likelihood of recurrence to a minimum. Increasing our knowledge based on cancer biogenesis and cancer treatment is fundamental to achieving such a goal. Currently, it is still unclear which combinations are the most suitable for the treatment of specific cancers, which is why we need to pursue research in this area.

## 7. Conclusions

TNBC is the most aggressive, metastatic and refractory subtype of BC, often occurring in younger patients with poor clinical prognosis. Given the current lack of specific targets for effective intervention, the development of an effective treatment strategy remains an unmet medical need. The increasing interest in EV-NDDS poses as an exciting novel avenue for cancer interventions, which seems to be promising in a multitude of cancer treatment; i.e., not limited to TNBC. Not only can EV-NDDS be manipulated for the delivery of endogenous or exogenous materials, but they can also be engineered in many ways to offer an even larger pool of therapeutic avenues, such as TAA-presenting EVs as a cancer vaccine. Streamlined strategies include isolating EVs from prescreened and characterized donor cells (manipulated or genetically modified; optional steps) cultured under GMP-compliant protocols, followed by EV isolation with or without loading strategies of the therapeutic agents of interest, and, finally, long-term stability of the EV therapeutic while maintaining its integrity and functionality. Ultimately, these EV-based therapeutic products are regarded as new off-the-shelf cell-free biologics. Moreover, TEVs playing prominent roles in metastasis and tumour progression can be repurposed as biomarkers for early detection and disease-progression monitoring. However, their mechanism and function in tumorigenesis have yet to be deeply investigated. There is still a lot to be elucidated about the role of TNBC-TEVs for diagnosis/prognosis and therapeutic purposes, especially in clinical studies. Further research will shed light on TNBC-TEVs’ pathogenesis, diagnosis/prognosis, EV-NDDS-based treatments and vaccines, chemoresistance and combinatorial therapy.

## Figures and Tables

**Table 3:** List of active and ongoing clinical trials investigating cancer vaccines for TNBC.

Trial (National Clinical Trial ID)	Phase	Condition	Interventions
NCT03362060	I	TNBC Metastatic TNBC	Drug: Pembrolizumab Biological: PVX-410
NCT02826434	I	BC	Biological: PVX-410 Biological: Durvalumab Drug: Hiltonol
NCT04105582	I	TNBC BC	Biological: Neo-antigen pulsed DCs
NCT02316457	I	TNBC	Biological: IVAC_W_bre1_uID Biological: IVAC_W_bre1_uID/IVAC_M_uID
NCT03199040	I	TNBC Metastatic TNBC	Drug: Durvalumab Biological: Neoantigen DNA vaccine Device: TDS-IM system (Inchor Medical Systems)
NCT04024800	II	TNBC	Biological: AE37 Peptide vaccine Biological: Pembrolizumab

Pembrolizumab: FDA-approved anti-PD-1 antagonist monoclonal antibody; PVX-410 and AE37: investigational peptide therapeutic as cancer vaccine. IVAC_W_bre1_uID: Individualized Cancer Immunotherapy patient-specific liposome complexed RNA tailored to the antigen-expression profile of any given patient’s tumour. IVAC_M_uID: Individualized Cancer Immunotherapy treatment with de novo synthesized RNAs targeting up to 20 individual tumour mutations. TDS-IM system: a dermal DNA vaccine delivery system.

**Table 4:** A comparison between EV-NDDS, synthetic NP-NDDS and naked drug delivery systems.

Component	EV-NDDS	Synthetic NP-NDDS	Naked Drug	Refs.
Inherent ability to cross the BBB and cross from the bloodstream into the brain.	Yes	No *	No	[46,47,48,49,201]
Susceptibility to the EPR effect (accumulation in tumour tissue).	Yes	Yes	No	[187,196,208]
Ability to cross cellular barrier.	Yes	Varied	Low	[208,209]
Improve intracellular penetration.	Yes	Yes	Varied	[205,210,211]
Targeted delivery (tissue or cell type specific) and co-delivery of multiple agents.	Yes	Yes	N/A	[46,69]
Application versatility (vaccine vehicle, immunotherapy, regenerative medicine, etc.).	Yes	Yes	N/A	[187,212]
Improved pharmacological properties such as solubility, in vivo stability (circulating half-life), pharmacokinetic profile, protection of biologic drugs from premature release and degradation.	Yes	Yes	N/A	[13,188,205,213]
Therapeutic index improvement either by increasing the efficacy or decreasing unwanted side effects.	Yes	Yes	N/A	[154,203]
Cargo diversity (nucleic acids, proteins, lipids, drugs, etc.).	Yes	Yes	N/A	[37,196]
Relative dosing.	Low	Low	High	[13,214]
Susceptibility to sheer force during nebulization, lyophilization and other extreme handling processes.	Low	Low	Low	[55,56,215]
Complexity of production, isolation and characterization.	Varied	Varied	Varied	[37,187,215,216,217]
Prevention of antidrug antibodies formation.	Varied	Varied	N/A	[62,191,203]
Responsiveness to the TME.	No	No	N/A	[37,218,219]
Environmental toxicity.	Low	Varied	Varied	[220,221]
Clinical toxicity.	Low	Varied	High	[154,222,223]
Potency after systemic delivery, biodistribution and biocompatibility.	High	Varied *	Low	[189,208,224]
Drug release versatility.	High	High	N/A	[203,225]
Engineering potential (composition, targeted delivery, selective packing, etc.).	High	High	N/A	[74,213,216]
Diagnostic potential.	Yes	Potentially	N/A	[44,203]
Ability to evade immune detection.	Varied	Varied	N/A	[41,226]
Potential to cause graft-vs.-host disease (GvHD).	Depends on producer cell	Unlikely	N/A	[227]
Intrinsic diversity.	High	Low	N/A	[37,187]

* Depending on surface coating.

## Abbreviations

APCs: Antigen-Presenting Cells; BBB: Blood–Brain Barrier; BC: Breast Cancer; BC-TEVs: Breast Cancer Tumour-Derived Extracellular Vesicles; CMC: Chemistry Manufacturing and Controls; CTLs: CD8+ Cytotoxic T Cells; CTL-EVs: CD8+ Cytotoxic T Cell-Derived Extracellular Vesicles; CAR: Chimeric Antigen Receptor; DCs: Dendritic Cells; DC-EVs: Dendritic Cell-Derived Extracellular Vesicles; DHMEQ: Dehydroxymethylepoxyquinomicin; DOX: Doxorubicin; ER: Estrogen Receptor; EPR: Enhanced Permeability and Retention; EVs: Extracellular Vesicles; EV-NDDS: Extracellular Vesicle Nanoscale Drug Delivery System; EGFR: Epidermal Growth Factor Receptor; FDA: Food and Drug Administration; GMP: Good Manufacturing Practices; GvHD: Graft-versus-Host Disease; HER2: Human Epidermal Growth Factor 2; HPV: Human Papillomavirus; Mab: Monoclonal antibody; miR: MicroRNA; MISEV: Minimal Information for Studies of Extracellular Vesicles; MHC-1: Major Histocompatibility Complex I; MPS: Mononuclear Phagocyte System; MSCs: Mesenchymal Stromal/Stem Cells; NDDS: Nanoscale Drug Delivery System; NK Cells: Natural Killer Cells; NK-EVs: Natural Killer Cell-Derived Extracellular Vesicles; NPs: Nanoparticles; NP-NDDS: Nanoparticle Nanoscale Drug Delivery System; PBMCs: Peripheral Blood Mononuclear Cells; PDX: Patient-Derived Xenografts; PEG: Polyethylene Glycol; PTX: Paclitaxel; PR: Progesterone Receptor; SEER: Surveillance, Epidemiology and End Results; siRNA: Small Interfering RNA; TAA: Tumour-Associated Antigen; TEVs: Tumour-Derived Extracellular Vesicles; TIL: Tumour-Infiltrating Lymphocytes; TIL-EVs: Tumour-Infiltrating Lymphocytes derived Extracellular Vesicles; TME: Tumour Microenvironment; TNBC: Triple-Negative Breast Cancer; TNBC-TEVs: Triple-Negative Breast Cancer Tumour-Derived Extracellular Vesicles; TROP2: Tumour-Associated Calcium Signal Transducer 2; UCHL1: Ubiquitin Carboxyl-Terminal Hydrolase Isozyme L1.
